# Improved assay for detecting SARS-CoV-2 from nonporous hospital surfaces using surrogate human coronavirus OC43

**DOI:** 10.1017/ash.2022.69

**Published:** 2022-05-16

**Authors:** Lisa Tran, William Furin, Geun Woo Park, Martinique Edwards, Carrie Whitworth

## Abstract

**Background:** Understanding SARS-CoV-2 persistence on surfaces can help inform transmission risk from surfaces in healthcare and community settings. A sensitive viral infectivity assay is crucial for the detection of infective virus in environmental investigations. The conventional cell culture-based infectivity assay is limited by the time dependence, subjectivity, and insensitivity of cytopathic effect (CPE) scoring. We validated an integrated cell-culture and reverse-transcription quantitative RT-PCR method (cc-RT-qPCR) to improve SARS-CoV-2 detection and reduce detection time. We compared cc-RT-qPCR with CPE-scored cell culture to evaluate assay sensitivity of recovered virus from stainless-steel coupons simulating nonporous healthcare surfaces. **Method:** Human β-coronavirus OC43, a model strain for SARS-CoV-2, was propagated on HRT-18G cells in growth medium at 33°C in a 5% CO_2_ incubator. The OC43 infectivity was determined by cell culture with a 10-fold dilution series of viral samples in 96-well plates, and incubation for 7 days at 33°C to confirm CPE. Plates were CPE-scored and TCID50 was calculated using the Reed-Muench method. For the cc-RT-qPCR assay, CPE-negative wells were interrogated for viral intracellular replication using RT-PCR; infectivity was based on a titer increase of ≥ 2 logs 7 days after inoculation using RT-qPCR. CPE-positive or replicative virus-harboring cells were enumerated to determine TCID_50_. The sensitivity of both CPE-scored cell culture and cc-RT-qPCR assays were evaluated by inoculating 105 TCID_50_/mL OC43 in infection media and artificial saliva matrices onto coupons and dried in an environmental chamber at 26°C and 57% relative humidity for 6 hours. Viral eluates from coupons served as test samples. **Results:** Low-titer infectious OC43 (0.75 log10) was detected by both methods 7 days after incubation; however, infectivity confirmation required 4 and 6 days after incubation, respectively, for cc-RT-qPCR and CPE-scored cell culture methods. When cells were inoculated with OC43 at titer range 1.75–4.75 log_10_, CPE presented at 4–5 days after incubation, while viral replication was already detected at 3 days after incubation via RT-PCR. Upon virus titration, cc-RT-qPCR demonstrated greater sensitivity, detecting up to 1 log_10_ higher of infectious OC43 than cell culture alone at 0 and 6 hours (*P* ≤ .05) dried in infection medium and 0 hours (*P* ≤ .05) in saliva. **Conclusions:** Our data demonstrated greater sensitivity and shorter times to detect viral replication by cc-RT-qPCR, minimizing potential for false-negative results with cell culture alone. This sensitive assay may provide investigators with quicker results for informing infection control practices to reduce risk of transmission from deposited bodily fluids on surfaces, eg, coughing and sneezing.

**Funding:** None

**Disclosures:** None

